# Case Report: Rare Presentation of Multivisceral Echinococcosis

**DOI:** 10.4269/ajtmh.18-0673

**Published:** 2019-03-11

**Authors:** Xiao Cai, Huixia Cai, Qing Gan, Wenxu Chang, Fang Yuan, Wei Luo, Jie Sun, Jing An

**Affiliations:** 1 Department of Otolaryngology Head and Neck Surgery, People’s Hospital of Qinghai Province, Xining, China;; 2 Department of Parasite Control, Qinghai Province Institute for Endemic Diseases Prevention and Control, Xining, China

## Abstract

Cystic echinococcosis (CE) is a common, chronic, and endemic zoonotic disease usually localized in a single organ; multivisceral cases are rare, especially outside the liver or lung. Here, we describe an unusual case of a 43-year-old Tibetan man with echinococcosis of the infratemporal fossa, heart, liver, pancreas, abdomen, and pelvic cavity. He only presented with diminished vision of the left eye, especially when chewing. Computed tomography and magnetic resonance imaging revealed multivisceral CE. The patient underwent surgery for the excision of a cyst in the infratemporal fossa, as well as chemotherapy, and the diagnosis was confirmed by histopathological examination. The diagnosis, clinical features, treatment, and follow-up in this case are discussed. In areas with high echinococcosis prevalence, examination by full imaging is necessary for an accurate diagnosis, especially in cases of atypical localization. Chemotherapy for treatment, as well as prophylaxis against recurrence, can be effective when surgery is not possible.

## INTRODUCTION

Echinococcosis caused by metacestodes of the genus *Echinococcus* is a chronic disease that is one of the most important zoonoses worldwide.^1^ It is a significant public health problem and economic burden, especially in pastoral areas of the western Tibetan region in China.^2^ Cystic echinococcosis (CE) is a type of echinococcosis caused by the parasite *Echinococcus granulosus*, which is widespread globally. In CE, cysts can occur at virtually any anatomical location, although the liver (70–80%) and lung (10–30%) are the most frequently affected organs, followed by other regions of the body (10–15%).^3–5^ Cases of multivisceral echinococcosis with atypical localization are very rare but can have severe consequences and may even be fatal. We present here the case of a male patient with cysts located in the infratemporal fossa, heart, liver, pancreas, abdomen, and pelvic cavity. The clinical manifestations, blood test results, imaging and histological findings, and treatment are discussed.

## CASE REPORT

**Figure 1.**
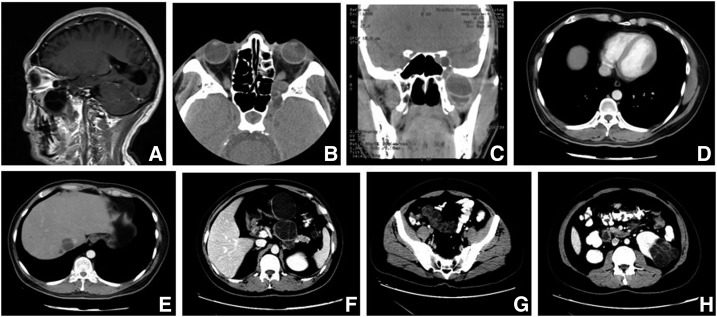
(**A**) Sagittal T2 magnetic resonance image showing the focal lesion with a complete edge and clear separation in the left infratemporal fossa. (**B** and **C**) Computed tomography (CT) images showing the cystic lesion infiltrating into the intracranial and orbital areas (CT values: 4 to 50 HU and 3 to 28 HU). (**D**) Contrast-enhanced CT image showing a low-density mass within the left ventricle (CT value: 12 to 27 HU). (**E**) Contrast-enhanced CT images showing a cystic lesion within the liver (CT value: −19 HU). (**F**) Contrast-enhanced CT image showing low-attenuation abdominal cystic masses (CT value: −6 to 18 HU) and a multiseptated mass within the pancreatic tail (CT value: −6 to 21 HU). (**G**) CT image showing an *Echinococcus granulosus* cyst containing daughter cysts in the pelvic cavity (CT value: −50 to 30 HU). (**H**) Contrast-enhanced CT image showing a multiseptated thick-walled cystic lesion in the left kidney (CT value: −24 to 27 HU).

A 43-year-old Tibetan man from the pastoral area in Qinghai, China, with a history of close contact with dogs and sheep, presented with diminished vision of the left eye, especially when chewing, which had persisted for over 1 month without any concomitant symptoms. The patient had been diagnosed with hepatic CE in 2010 but had not received any treatment at the time. Magnetic resonance imaging (MRI) and computed tomography (CT) of the paranasal sinus revealed a 47 × 44-mm cystic mass in the left infratemporal fossa, which was determined as type CE2 according to the WHO classification. Some lesions infiltrated into the intracranial and orbital areas, and the upper maxillary sinus cavity was compressed, as evidenced by contrast-enhanced MRI (Figure 1A–C). A transthoracic echocardiogram showed a rounded cyst measuring 16 × 20 mm with a clear boundary, regular shape, and homogenous hypoecho in the left ventricle myocardium. Left ventricle systolic function was normal (left ventricular ejection fraction of 66%). Contrast-enhanced chest CT revealed a low-density mass measuring approximately 33.05 × 12.27 mm within the left ventricle (Figure 1D). An upper abdominal CT scan revealed two round, low-density, echogenic, cystic lesions involving segment seven of the liver, with the largest lesions measuring approximately 28.09 × 27.32 mm; multiple small vesicular structures were also visible within the lesions and were determined to be WHO type CE2.^3^ Small lesions were also visible beside the large one (Figure 1E). We also detected multiple purely unilocular cystic low-attenuation masses in the abdomen, with the largest lesions being type CE1 and measuring approximately 60.44 × 54.40 mm, and a low-attenuation multiseptated mass of type CE2 in the pancreatic tail measuring approximately 44.96 × 51.43 mm (Figure 1F). A pelvic CT scan showed uneven distribution of intracystic density and the presence of WHO type CE3B daughter cysts measuring 92.02 × 37.21 mm in diameter located in the pelvic cavity, as well as a large, multiseptated cystic mass of type CE2 measuring 62.32 × 62.51 mm in the left kidney. In addition, the patient was positive for the presence of IgG antibodies against *E. granulosus* cyst fluid antigen, which was detected using a commercial kit (Haitai Biological Pharmaceuticals Co., Zhuhai, China).

**Figure 2.**
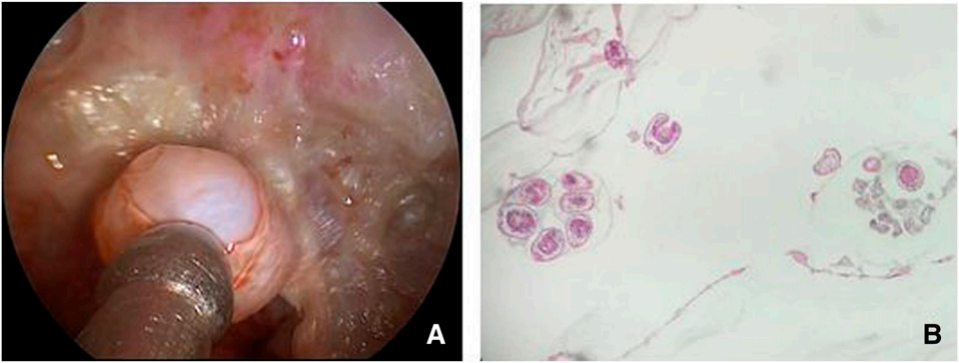
(**A**) Nasal endoscope aspect of the cystic lesion in the infratemporal fossa. (**B**) Histopathological analysis of a specimen by hematoxylin and eosin staining; a cystic mass with protoscoleces and laminated layers can be seen (original magnification: ×10). This figure appears in color at www.ajtmh.org.

The patient was treated with preoperative albendazole for 2 weeks (15 mg/kg/day). In light of the patient’s obvious eye symptoms, we decided to proceed with surgery for CE of the infratemporal fossa. Under general anesthesia, the cyst was identified and exposed in the lateral wall of the maxillary sinus (Figure 2A) and was aspirated with a large bore needle to remove the hydatid fluid before injection of a concentrated iodine solution for 10 minutes. The large cyst and its daughter cysts were completely eliminated. The cyst wall was then dissected from the adjacent tissue using both blunt and sharp dissection techniques. Pathological examination of the specimen revealed irregular laminated layers and protoscoleces (Figure 2B).

**Figure 3.**
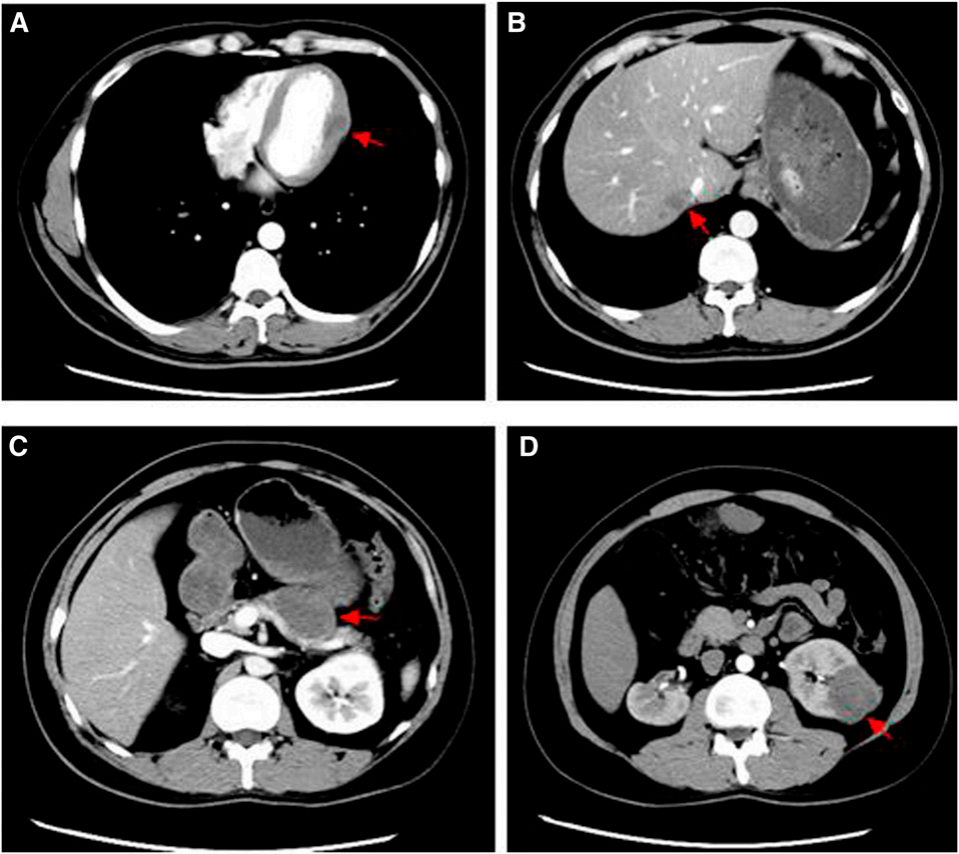
(**A**–**D**) Posttreatment computed tomography (CT) images of the left ventricle (CT value: 26 to 40 HU) (**A**), liver (CT value: 27 to 49 HU) (**B**), pancreatic tail (CT value: 26 to 51 HU) (**C**), and left kidney (CT value: 19 to 49 HU) (**D**). This figure appears in color at www.ajtmh.org.

After the operation, the patient recovered good eyesight and the lower nasal passage was unobstructed. Dynamic endoscopy showed that the mucosa in the cavity was smooth and without abnormalities 2 weeks after the operation. To treat echinococcosis cysts in the other organs and prevent a relapse requiring operation, the patient continued to take albendazole (15 mg/kg/day, 3-week treatments separated by 1-week intervals). After 16 months of follow-up, head, chest, abdominal, and pelvic CT scans showed no evidence of relapse and the left maxillary sinus structure had returned to normal. Surprisingly, the cysts in the abdomen and pelvic cavity had disappeared. The lesions in the left ventricle, liver, tail of the pancreas, and left kidney had decreased in size; intracystic tension was diminished; and the density was higher, with CT values of 26–40 HU, 27–49 HU, 26–51 HU, and 19–49 HU, respectively. Multiseptated cysts had disappeared completely or were almost undetectable in the liver and tail of the pancreas and left kidney (Figure 3A–D) as compared with pretreatment (Figure 1E, F, and H). We recommended that oral albendazole be continued, with regular follow-ups. The postoperative course was satisfactory, and no recurrence or abnormalities occurred at the surgical site.

## DISCUSSION

Cystic echinococcosis is a widespread chronic endemic helminthic disease caused by infection with metacestodes (larval stage) of the tapeworm *E. granulosus*. Transmission is mainly among dogs and livestock (typically, a dog–sheep–dog cycle). Dogs are the definitive hosts for this parasite. Eggs in dog feces contaminate vegetation in meadows, which is ingested by the intermediate hosts (e.g., sheep and yak). Humans are an accidental intermediate host for this parasite and are normally infected by the ingestion of eggs released from dogs.^2,6^ Most infected individuals are asymptomatic; CE is characterized by slowly enlarging cysts that often grow unnoticed and are neglected for years until acute complications occur.^7,8^ Echinococcosis is highly endemic in 39 of 43 counties of Qinghai Province, with an estimated prevalence of 0.63% (range: 0.03–12.38%).^9^ Our patient was from Yushu Prefecture in the Qinghai southern plateau, which has an estimated CE prevalence of 4.54%.^10^ The patient had a history of close contact with dogs and sheep, which made him susceptible to the disease.

Multivisceral echinococcosis, the simultaneous localization of hydatid cysts in more than one organ, is rarer than multiple echinococcosiswherein multiple hydatid cysts occur in the same organ.^5^ Multivisceral echinococcosis of the abdomen is relatively common,^4,11^ accounting for more than 5% of hospitalized cases in endemic areas of China.^12,13^ However, multivisceral echinococcosis at unusual anatomical locations, as in our patient, is rare—that is, simultaneous localization of hydatid cysts in as many as six organs. Echinococcosis mostly involves two or three organs in multivisceral cases reported. Moreover, our patient had many hydatid cysts at unusual locations, such as the infratemporal fossa, pancreas, and left ventricle; in particular, localization in the infratemporal fossa has never been reported. This represents a more serious form of echinococcosis that differs from cases of multiple hydatid cysts in the same organ or mono-visceral echinococcosis.^5^ Computed tomography scanning has many advantages for the diagnosis of multivisceral echinococcosis and postoperative surveillance.^4,5,14^ The main treatment for echinococcosis is surgery. However, chemotherapy with albendazole, benzimidazole, or mebendazole can be effective perioperatively or between serial surgeries to sterilize the cyst and reduce the risk of anaphylaxis or recurrence^15–17^ and as maintenance therapy for patients who are excluded from surgery. The cysts of our patient were mainly classified as the CE2 type, which is more active and usually contains daughter cysts and living protoscoleces. However, after medical treatment, the cysts disappeared or were reduced in size, and intracystic density increased as compared with that before treatment, indicating that the activity of the parasite had declined; thus, the treatment was more effective than expected. This was attributed to good medication compliance by the patient and the efficacy of albendazole against CE.

Not all patients with multivisceral echinococcosis have obvious clinical symptoms. About 10–30% of patients with echinococcosis have clinical manifestations^18^; the cyst size, location, space occupied, and degree of oppression of adjacent tissues or organs can indicate whether the disease has advanced to the middle or late stage. In our patient, the major complaint was diminished vision in the left eye, especially when chewing, resulting from the penetration of the cystic lesion into the intracranial and orbital areas close to the optic nerve. When the patient chewed, the lesion further compressed the optic nerve, leading to obvious clinical symptoms in the left eye.

Routine imaging and detection of the typical imaging features of echinococcosis are important for diagnosing the disease.

## CONCLUSION

Imaging is critical for the diagnosis of multivisceral echinococcosis, especially in cases of atypical localization. In our case, a combination of local lesion resection and chemotherapy to control the disease achieved a relatively satisfactory outcome. Although not all the cysts were completely removed, the treatment regimen was more acceptable to the patient than full surgery. Thus, chemotherapy is a suitable option when a patient refuses or is unable to undergo surgery.
